# Effectiveness of the Common Elements Treatment Approach (CETA) in reducing intimate partner violence and hazardous alcohol use in Zambia (VATU): A randomized controlled trial

**DOI:** 10.1371/journal.pmed.1003056

**Published:** 2020-04-17

**Authors:** Laura K. Murray, Jeremy C. Kane, Nancy Glass, Stephanie Skavenski van Wyk, Flor Melendez, Ravi Paul, Carla Kmett Danielson, Sarah M. Murray, John Mayeya, Francis Simenda, Paul Bolton

**Affiliations:** 1 Department of Mental Health, Johns Hopkins Bloomberg School of Public Health, Baltimore, Maryland, United States of America; 2 Department of Epidemiology, Columbia University Mailman School of Public Health, New York, New York, United States of America; 3 Johns Hopkins University School of Nursing, Baltimore, Maryland, United States of America; 4 Department of Psychiatry, School of Medicine, University of Zambia, University Teaching Hospital, Lusaka, Zambia; 5 National Crime Victims Research and Treatment Center, Department of Psychiatry and Behavioral Sciences, Medical University of South Carolina, Charleston, South Carolina, United States of America; 6 Ministry of Health–Zambia, Chainama Hills College Hospital, Lusaka, Zambia; 7 Chainama Hills College Hospital, Lusaka, Zambia; 8 Department of International Health, Johns Hopkins Bloomberg School of Public Health, Baltimore, Maryland, United States of America; University of New South Wales, AUSTRALIA

## Abstract

**Background:**

Both intimate partner violence (IPV) and alcohol misuse are highly prevalent, and partner alcohol misuse is a significant contributor to women’s risk for IPV. There are few evidence-based interventions to address these problems in low- and middle-income countries (LMICs). We evaluated the effectiveness of an evidence-based, multi-problem, flexible, transdiagnostic intervention, the Common Elements Treatment Approach (CETA) in reducing (a) women’s experience of IPV and (b) their male partner’s alcohol misuse among couples in urban Zambia.

**Methods and findings:**

This was a single-blind, parallel-assignment randomized controlled trial in Lusaka, Zambia. Women who reported moderate or higher levels of IPV and their male partners with hazardous alcohol use were enrolled as a couple and randomized to CETA or treatment as usual plus safety checks (TAU-Plus). The primary outcome, IPV, was assessed by the Severity of Violence Against Women Scale (SVAWS) physical/sexual violence subscale, and the secondary outcome, male alcohol misuse, by the Alcohol Use Disorders Identification Test (AUDIT). Assessors were blinded. Analyses were intent-to-treat. Primary outcome assessments were planned at post-treatment, 12 months post-baseline, and 24 months post-baseline. Enrollment was conducted between May 23, 2016, and December 17, 2016. In total, 123 couples were randomized to CETA, 125 to TAU-Plus. The majority of female (66%) and a plurality of male (48%) participants were between 18 and 35 years of age. Mean reduction in IPV (via SVAWS subscale score) at 12 months post-baseline was statistically significantly greater among women who received CETA compared to women who received TAU-Plus (−8.2, 95% CI −14.9 to −1.5, *p =* 0.02, Cohen’s *d* effect size = 0.49). Similarly, mean reduction in AUDIT score at 12 months post-baseline was statistically significantly greater among men who received CETA compared to men who received TAU (−4.5, 95% CI −6.9 to −2.2, *p* < 0.001, Cohen’s *d* effect size = 0.43). The Data and Safety Monitoring Board recommended the trial be stopped early due to treatment effectiveness following the 12-month post-baseline assessment, and CETA was offered to control participants. Limitations of the trial included the lack of a true control condition (i.e., that received no intervention), self-reported outcomes that may be subject to social desirability bias, and low statistical power for secondary IPV outcomes.

**Conclusions:**

Results showed that CETA was more effective than TAU-Plus in reducing IPV and hazardous alcohol use among high-risk couples in Zambia. Future research and programming should include tertiary prevention approaches to IPV, such as CETA, rather than offering only community mobilization and primary prevention.

**Trial registration:**

The trial was registered on ClinicalTrials.gov (NCT02790827).

## Author summary

**Why was this study done?:**

- Intimate partner violence (IPV) experienced by women is a highly prevalent global public health concern that is associated with substantial morbidity and mortality.
- In many low- and middle-income countries (LMICs), such as Zambia, there is a lack of evidence-based intervention approaches to prevent and reduce IPV.
- One key risk factor for a woman experiencing IPV is when her male partner has unhealthy alcohol use.
- We previously found that an evidence-based psychotherapy intervention delivered by lay counselors in LMICs (known as the Common Elements Treatment Approach [CETA]) was effective in reducing mental and behavioral health problems among violence-affected populations.

**What did the researchers do and find?:**

- We conducted a randomized controlled trial to evaluate the effectiveness of CETA in reducing IPV and hazardous alcohol use among couples in Zambia.
- We recruited heterosexual couples in which the woman reported experiencing recent IPV that was perpetrated by her current male partner and in which the man had recent hazardous alcohol use.
- Compared to usual care plus safety checks, our results showed that, 1 year following treatment commencement, CETA was clinically effective in reducing IPV experienced by women and men’s hazardous alcohol use.

**What do these findings mean?:**

- The findings suggest that a clinical psychotherapy treatment is one effective tool for reducing IPV in resource-limited settings.
- Given that IPV is such a complex social problem, future evaluations would benefit from combining primary, secondary, and tertiary prevention approaches to enhance effectiveness, such as combining CETA with effective economic interventions and potentially higher level psychological and/or psychiatric care when available and indicated.

## Introduction

Intimate partner violence (IPV) is a highly prevalent global health and human rights concern [1–3]. IPV encompasses physical, sexual, psychological, and emotional violence and abuse within an intimate relationship that causes or has the potential to cause physical, sexual, or psychological harm [4]. Global estimates indicate that approximately 1 in 3 women and girls who have been in a relationship have experienced physical or sexual violence by a partner or ex-partner [5]. In Zambia, the site of the present study, analysis of the 2013–2014 Demographic and Health Survey found a higher rate than global estimates, with 43% of women aged 15–49 years reporting lifetime experience of physical and/or sexual IPV and 27% of women reporting physical and/or sexual IPV in the past 12 months [6].

International studies have repeatedly demonstrated the adverse health, economic, and social effects of IPV on individuals, couples, and communities. Health effects range from immediate impact, such as injury and infection (e.g., sexually transmitted infection/HIV), to long-term sequelae of substance use, depression, anxiety, or other related mental problems, and unplanned pregnancy and/or poor pregnancy outcomes (e.g., low birth weight) [7–13]. Children that grow up in violent homes have an increased risk of being victims or perpetrators of violence in their own adult intimate relationships [14,15]. The economic and societal impacts of IPV are related to lost wages, lower productivity, and increased health expenditures and costs from resources utilized [16]. For low- and middle-income countries (LMICs), such as in Zambia, addressing IPV is a critical target for sustainable development [17].

Despite the substantial burden of violence in LMICs [1] and the link between IPV and mental/behavioral health (e.g., depression, trauma, alcohol misuse) [7], there is limited evidence of effective interventions for IPV in low-resource contexts. In particular, there has been a dearth of attention in addressing the health and treatment needs of the male perpetrators of IPV, especially related to mental health and alcohol misuse [18–20]. This is problematic because male partner alcohol misuse can significantly increase the risk of women experiencing IPV [21]. Two trials conducted in the US suggest cognitive behavioral therapy (CBT) interventions and alcohol treatment combined with violence prevention programs show promise for impacting some types of IPV [22,23]. Therefore, evaluation of interventions that (a) include both women who have experienced IPV and their male partners who perpetrate the violence and (b) target the violence by addressing its underlying causes, such as alcohol misuse, are urgently needed.

The primary aims of this community-based randomized controlled trial (RCT) are to evaluate the effectiveness of an evidence-based, multi-problem, flexible, transdiagnostic intervention, the Common Elements Treatment Approach (CETA) [24], with couples living in urban Zambia on (a) reducing and preventing women’s experience of IPV and (b) reducing male partner’s hazardous alcohol use. The study utilized a secondary prevention approach, which by definition mitigates harm within a sample currently experiencing the condition, in this case IPV. This trial is part of the global research consortium What Works to Prevent Violence (https://www.whatworks.co.za/).

## Methods

### Study design

This was a single-blind, parallel-assignment RCT. The trial protocol was submitted to ClinicalTrials.gov in early May 2016, before study commencement; following an administrative review, the record was released from the investigator’s institution on May 24, 2016, and following quality control and administrative changes, it was publicly released on June 6, 2016 (NCT02790827). The protocol was also previously published in the peer-reviewed literature [25].

The trial was conducted in 3 high-density, low-socioeconomic neighborhoods (“compounds”), in Lusaka, the capital city of Zambia. The compounds are around 8 kilometers from each other, with the furthest two being about 16 km from each other, and with populations ranging from approximately 30,000 to 60,000. Study recruitment, intervention delivery, and outcome assessments were all conducted in community locations (e.g., community centers, schools, churches) within these compounds.

### Ethical approval

Ethical approval was obtained from the Johns Hopkins Bloomberg School of Public Health Institutional Review Board and the University of Zambia Biomedical Ethics Review Committee. Informed oral consent was obtained from all participants.

### Participants

#### Recruitment

Community-based recruitment of participants was conducted by study counselors, in accordance with how a CETA program would most likely be implemented outside the context of a clinical trial. Prior to study commencement, informational meetings about the study were held for community members in each of the 3 sites that included time for individuals to ask questions and provide feedback [26,27]. Following a 1-day training on human participants and participant recruitment, CETA counselors in male–female pairs went door to door in their own communities and met privately with couples (women and men who were married, dating, or in a relationship) to provide more information about the study. Following a brief, standardized script, the CETA counselors explained that there was a study offering help to address IPV and alcohol misuse and asked if the male or female would like more information. For safety and ethical reasons, the female counselor spoke privately with the adult female in the home to assess immediate health and/or safety risks for her or her children. If there were risks, counselors provided information on accessing relevant services and offered to assist the woman and/or her children (if applicable) in safety planning or reporting, such as by taking them to the Zambia Police Victim Support Unit.

Couples who expressed interest in learning more about the study were provided with the research team’s contact information and were pre-scheduled to attend a meeting with a study research assistant (typically within 1 week of the recruitment contact).

#### Screening and baseline assessment

The screening and assessment activities were conducted by a research assistant in a private area of a community location (e.g., church or school). Informed oral consent was obtained separately for each family member by the research assistant in the language of the participant’s choice (English, Nyanja, or Bemba). In order to be eligible, (a) the couple had to be living in 1 of the 3 study neighborhoods in Lusaka; (b) the couple had to speak English, Bemba, or Nyanja; (c) the couple had to both be 18 years of age or older; (d) the adult female had to report at least moderate levels of past-year physical/sexual IPV perpetrated by her male partner (indicated by a score of 38 or higher on the Severity of Violence Against Women Scale [SVAWS] physical/sexual violence subscale); (e) the adult male had to self-report, or the female had to partner-report, that the male had hazardous alcohol use as indicated by a score of 8 or higher on the Alcohol Use Disorders Identification Test (AUDIT). Exclusion criteria were (a) recent suicide attempt or ideation with specific intent, plan, or self-harm; (b) a diagnosed psychotic disorder; (c) a severe developmental disorder; or (d) currently on an unstable psychiatric drug regimen.

The man and the woman within a couple were screened for eligibility separately on laptops using audio computer-assisted self-interviewing (ACASI). The use of ACASI, which we previously piloted and tested in Zambia [28], permitted the participants to self-complete the questionnaires privately and without the presence of a face-to-face interviewer. If a participant experienced any technical difficulty in using ACASI or had a question, a research assistant was nearby to assist. For women, the eligibility screen included demographic items, SVAWS, and the AUDIT partner-report. The adult male eligibility screen included demographic items and a self-reported AUDIT.

Immediately after the participants finished the screening, ACASI provided the research assistant with eligibility status. Ineligible participants were thanked for their time and provided a list of relevant services. Individuals found to be eligible completed the full set of outcome questionnaires using ACASI (see “Outcomes”).

### Randomization and masking

Eligible couples were randomized as a unit 1:1 to either CETA or treatment as usual plus safety checks (TAU-Plus). Randomization was conducted off-site in the US by an investigator who had no interaction with participants. Three lists (1 for each study site) were maintained, each with random treatment assignments to CETA and TAU-Plus in blocks of 20 (i.e., for every 20 assignments in random order, 10 were CETA and 10 were TAU-Plus). Microsoft Excel random number generator was used to create the random sequence. The randomization list was password protected and securely stored on a separate server from all other study materials and documents. No study staff based in Zambia had access to the list or knowledge of the randomization blocking (blocks of 20) or stratified (by site) design. Eligible couple ID numbers were forwarded from the field site in Zambia to the US within 1 day of completing the screening. The ID numbers were allocated to the next available slot in the randomization list, and the Zambia-based study director was informed about the result of the randomization. Participants were contacted by the Zambia study team within a week of consent and eligibility screening, and told of their treatment assignment. Couples who were randomized to CETA were contacted by a CETA counselor within a week of their baseline assessment. Although couples were randomized as a unit, there were separate treatment sessions for men and women.

Research assistants who conducted outcome assessments were masked to the participant’s treatment assignment, as was the data analyst. Due to the nature of the CETA intervention and TAU-Plus control condition, study participants and counselors were not masked. Participant treatment assignment was maintained in a separate database from outcome data and was only merged during the final step in analysis of trial outcomes.

### Procedures

CETA is a cognitive-behavioral, modular, flexible, multi-problem, transdiagnostic treatment model that was developed based on advances in high-income settings and built specifically for implementation in LMICs with lay providers [24]. CETA is not conceptualized as a “new treatment” but rather an approach to teaching CBT skills that allows for more effective, efficient, and economic scale-up and sustainability. It specifically addresses the issue of comorbidity in mental and behavioral health, which is the rule, not the exception [29]. CETA comprises 9 evidence-based, widely used CBT elements: engagement, introduction/psychoeducation, safety, substance use reduction, cognitive coping and restructuring, problem solving, behavioral activation, relaxation, and exposure (live and imaginal). CETA teaches decision rules on which elements to provide based on research evidence generated worldwide, but permits flexibility, to address comorbidity and individualized treatment. CETA is unique in that (a) it is built specifically for lower-income settings and delivery by lay providers, (b) it addresses multiple problems such as trauma, violence, anxiety, depression, functioning, and behavioral problems for youth, (c) it utilizes steps sheets for stronger fidelity, and (d) it has shown strong effectiveness in multiple randomized clinical trials. On the Thailand/Myanmar border, CETA’s effect sizes (Cohen’s *d*) were moderate to large for improving depression, post-traumatic stress, impaired function, anxiety, and aggression (range *d =* 0.58–1.19) [30]. In Iraq, there were large effect sizes for improving trauma, anxiety, and depression (range *d =* 1.56–2.38) [31]. CETA has been described elsewhere in greater detail (https://www.cetaglobal.org) [24]. CETA has also shown effectiveness in reducing internalizing symptoms, externalizing behaviors, and post-traumatic stress, and improving well-being, among Somali refugee youth in Ethiopia in an open trial [32].

CETA was modified to address IPV and alcohol/substance misuse, which is fully described in the previously published protocol paper [25]. Briefly, 2 elements were added to CETA: one on substance use and one on safety for violence. The substance use element was developed from evidence-based models (e.g., Risk Reduction through Family Therapy [33] and Relapse Prevention [34]) and was adapted to fit within CETA by 2 authors (CKD, LKM). It was designed to be delivered in approximately 2 sessions with ongoing check-ins. An additional 1-session “substance use support” session was designed to allow for spousal support, as research has shown that family involvement can improve substance abuse outcomes during and after treatment [35]. The safety for violence element was specific to safety planning, utilizing a problem-solving framework in order to complement the existing safety element in CETA (direct behavioral safety planning). CETA was also modified to be delivered in group format, with separate groups for men and women.

CETA providers were lay counselors, or individuals with no formal mental health training, (20 male, 43 female), plus 7 supervisors (3 male, 4 female) between the ages of 20 and 60 years (average age 33.7 years). The apprenticeship model of training and supervision was utilized, which includes a 10-day in-person training, followed by weekly small group meetings to practice the elements via role plays with other providers [36]. Each week the supervisors had a call with a CETA trainer to review the practice agenda and continue building capacity. CETA providers continued to meet in small groups and receive local supervision on each case throughout the study. The supervisor had weekly oversight from a CETA trainer. Fidelity was tracked utilizing Excel logs, with objective documentation of the steps completed in each element, as well as overall skill rating systems.

The control condition, TAU-Plus, was defined by our trained study team. There is no standard of care in Zambia for IPV or alcohol misuse; however, we provided couples with the contact information of existing community-based services that offer informal counseling at local organizations in Lusaka. Due to the high-risk nature of our study population, we determined that it was ethically imperative to conduct regular safety check-ins with control participants. We trained study research assessors (not otherwise clinically trained) on risk assessment and implementation of specific safety procedures for suicide, homicide, child abuse, and IPV. During the “treatment phase” of the study (the first 12 weeks following baseline), the assessors conducted weekly check-ins by phone. If participants were unreachable, the assessors would follow up with a home visit. During the check-ins, assessors asked all participants 4 questions on suicidal and homicidal ideation, and current risk of IPV and child abuse. If there was risk, a trained clinical supervisor was contacted, and a safety plan was created. The safety protocol has been described previously [37] and is included in the previous protocol publication [25].

Following the treatment phase, both CETA and TAU-Plus participants received monthly phone check-ins following the same process outlined above to assess safety. Safety checks were also completed at every outcome assessment.

### Outcomes

Outcomes were planned to be assessed via ACASI at baseline and 3 post-baseline timepoints: (a) within 1 month of treatment completion (i.e., approximately 3–4 months post-baseline), (b) 12 months post-baseline, and (c) 24 months post-baseline. The primary outcome was female self-report of IPV assessed by the SVAWS physical/sexual violence subscale [38]. SVAWS includes items focusing on physical violence (21 items), sexual violence (6 items), and threats of violence (19 items). For our eligibility screening and primary IPV outcome, we combined the physical and sexual violence items into 1 scale (27 items), as has been done previously among couples experiencing violence and alcohol abuse in South Africa [39]. In response to these items, women indicated how often they experienced each physical or sexual IPV event using a Likert-type scale in the past 12 months (for baseline, 12-month post-baseline, and 24- month post-baseline visits) or in the past 3 months (for the post-treatment visit). At baseline, scores could range from 38 (the minimum score for inclusion) to 108. We analyzed the threatened violence subscale separately (19 items).

In order to facilitate comparisons across studies within the What Works to Prevent Violence consortium of studies, all trials used a common IPV measure derived from the World Health Organization (WHO) Multi-Country Study on Women’s Health and Domestic Violence against Women [40]. The WHO-derived measure included 9 items in total (6 physical IPV and 3 sexual IPV). Each asks a woman about how often the experience occurred (never, once, a few times, many times). The reference periods used were the same as for SVAWS. Additionally, the scale was modified so that the items referred to perpetration (rather than experience) of the 9 types of violence, and these items were administered to male participants. Four binary variables were derived from this scale to use in analysis. A variable of “any physical IPV” was derived as a 1 (if the woman reported that she experienced at least 1 of the 6 physical IPV items once or more) or 0 (if she reported never experiencing any of the 6 items). A similar “any sexual IPV” binary variable was derived from the 3 sexual IPV items. The same 2 variables were derived with respect to perpetration of physical and sexual IPV from the male reports.

Alcohol use, a secondary outcome, was measured with AUDIT [41]. AUDIT is one of the most widely used tools for measuring alcohol consumption and related harms and was validated previously in Zambia [42]. The tool includes 10 items that ask about frequency and quantity of use, binge drinking, abuse and dependence symptoms, and consequences of use. Scores can range from 0 to 40; scores ≥8 among men are considered to indicate hazardous alcohol use [43]. In this study, we asked study participants to complete 2 versions of AUDIT: a self-report (about their own drinking) and a partner-report (in reference to their partner’s drinking).

This primary trial paper reports on the IPV outcomes (SVAWS physical/sexual violence subscale, SVAWS threatened violence subscale, WHO any physical violence binary item, WHO any sexual violence binary item) and alcohol outcomes (AUDIT scores for self and partner). The study also captured a wide range of secondary outcomes from adult and child participants that will be reported in subsequent papers. Secondary adult outcomes included depression, trauma, other substance use, psychological abuse, and gender norms. Secondary child outcomes included internalizing symptoms, externalizing behaviors, trauma, substance use, victimization, and aggression [25].

### Statistical analysis

The sample size calculation for the trial was based on a study by Peltzer and Pengpid [39] that used the SVAWS physical/sexual violence subscale to assess IPV among women whose male partners had alcohol use problems. We assumed a baseline mean on the subscale of 58.6 (SD = 20.5). The calculation was then based on an expectation of finding a mean reduction of 11.66 (20% reduction) in SVAWS score among women randomized to CETA and no change among women who were randomized to TAU-Plus. Specifying 80% power and an alpha level of 0.05, we calculated a minimum necessary sample size of 50 couples per arm. The sample size was increased to 84 couples per arm (168 total) to account for any possible small clustering effects attributable at the counselor level (assuming ICC = 0.1) and potential loss to follow-up/drop-out (20%).

Primary analyses were intent-to-treat and included all enrolled participants. Multiple imputation with chained equations was used to address missing data [44]. All timepoints were included in the imputation procedure, and imputed variables included the outcomes of interest, the SVAWS subscale score, the AUDIT score, and the WHO IPV items. We followed recommendations by White and colleagues of performing a number of imputations approximately equivalent to the amount of missing data; we therefore imputed 17 datasets [45].

Mixed effects regression models were estimated separately for each outcome (linear or generalized linear depending on the nature of the outcome). Fixed effects in the models included treatment group, time, and a group by time interaction. The group × time interaction was the primary coefficient of interest and represents the difference in mean change from baseline to follow-up between the CETA and TAU-Plus groups (for the linear models) and the ratio of relative risks (RRs) (for the generalized linear models). Additional demographic variables were considered for inclusion in the models as covariates if there was a substantial difference in the variable between treatment groups at baseline or if the variable predicted change in the outcome over time. To test the latter, we estimated separate mixed effects regression models to test whether demographic variables (age, ethnicity, education, employment, income, relationship status, disability status, HIV status, and number of trauma types experienced) predicted change in each outcome over time. Separate models were estimated for each combination of outcome (SVAWS, WHO IPV, or AUDIT) and demographic variable. The models included a possible demographic predictor of outcome change (e.g., age), time, and an interaction between the predictor of interest and time. Variables that predicted change in the outcome over time, as evidenced by statistical significance of the interaction term (*p* < 0.05), were subsequently included as a covariate in the main treatment effects model for that outcome. Variables included in each model are listed in S1 Table. We also conducted a sensitivity analysis in which the final models included the baseline value of the outcome as a fixed effect.

Participant ID and counselor ID were included as random effects to account for repeat measures of the same individual over time and clustering by counselor; as TAU-Plus participants did not receive counseling, they were assigned a dummy counselor ID code for analysis. Robust standard errors were estimated using the *vce(robust)* option in Stata [46]. This method, also known as a “sandwich” variance estimator, follows methods first described by Huber [47] and White [48]. For continuous outcomes, Cohen’s *d* effect sizes, equivalent to *Z*-scores of a standard normal distribution, were calculated as the group × time interaction term (difference in mean change from baseline to follow-up between the CETA and TAU-Plus groups) divided by the pooled baseline standard deviation. Interpretation of effect sizes was as follows: 0.2 represented a small clinical treatment effect, 0.5 a medium effect, and 0.8 or above a large effect [49]. For binary outcomes, RRs were calculated from the models, as was the ratio of RRs (group × time interaction term from the generalized linear models). Analyses were conducted using Stata 15 [46].

### Protocol changes

It became evident shortly after commencement of the trial that group-delivered CETA would be infeasible for our target population in urban Zambia. Participants had difficulty attending group therapy sessions at the same time due to conflicting schedules, unreliable transport in an urban setting (e.g., breakdown of a minibus), and conflicting livelihood or community activities (e.g., childcare, funerals, or job opportunities). Counselors had to conduct individual make-up sessions for multiple members in each group each week, leading to increased transport costs and provider time. Based on these challenges, we changed the mode of CETA delivery from group to individual counseling. In order to have the ability to conduct a sensitivity sub-analysis with participants who received only individual CETA counseling (excluding those who received any group therapy) compared to control, an additional cohort of *n =* 80 couples was recruited and randomized to CETA or TAU-Plus using the same methods as previously described. The total final sample size for the study was *n =* 248 couples.

### Data and safety monitoring

The trial was monitored by a 4-person Data and Safety Monitoring Board (DSMB) composed of experts on IPV, alcohol misuse, and randomized trials that were based in both sub-Saharan Africa and the US, including 1 author (NG). At the commencement of the study, the DSMB and study investigators agreed on stopping rules due to the high-risk characteristics of the study population. Specifically, an effectiveness analysis was planned following the 12-month post-baseline assessment with all available (i.e., non-imputed) data. If CETA was found to be clinically (Cohen’s *d ≥* 0.5) and statistically (*p* < 0.05) more effective than TAU-Plus in reducing SVAWS physical/sexual violence subscale score, then the trial would be stopped early and CETA would be provided to control participants. In that scenario, only the original CETA participants would be followed for an additional assessment at 24 months post-baseline.

## Results

**Fig 1:**
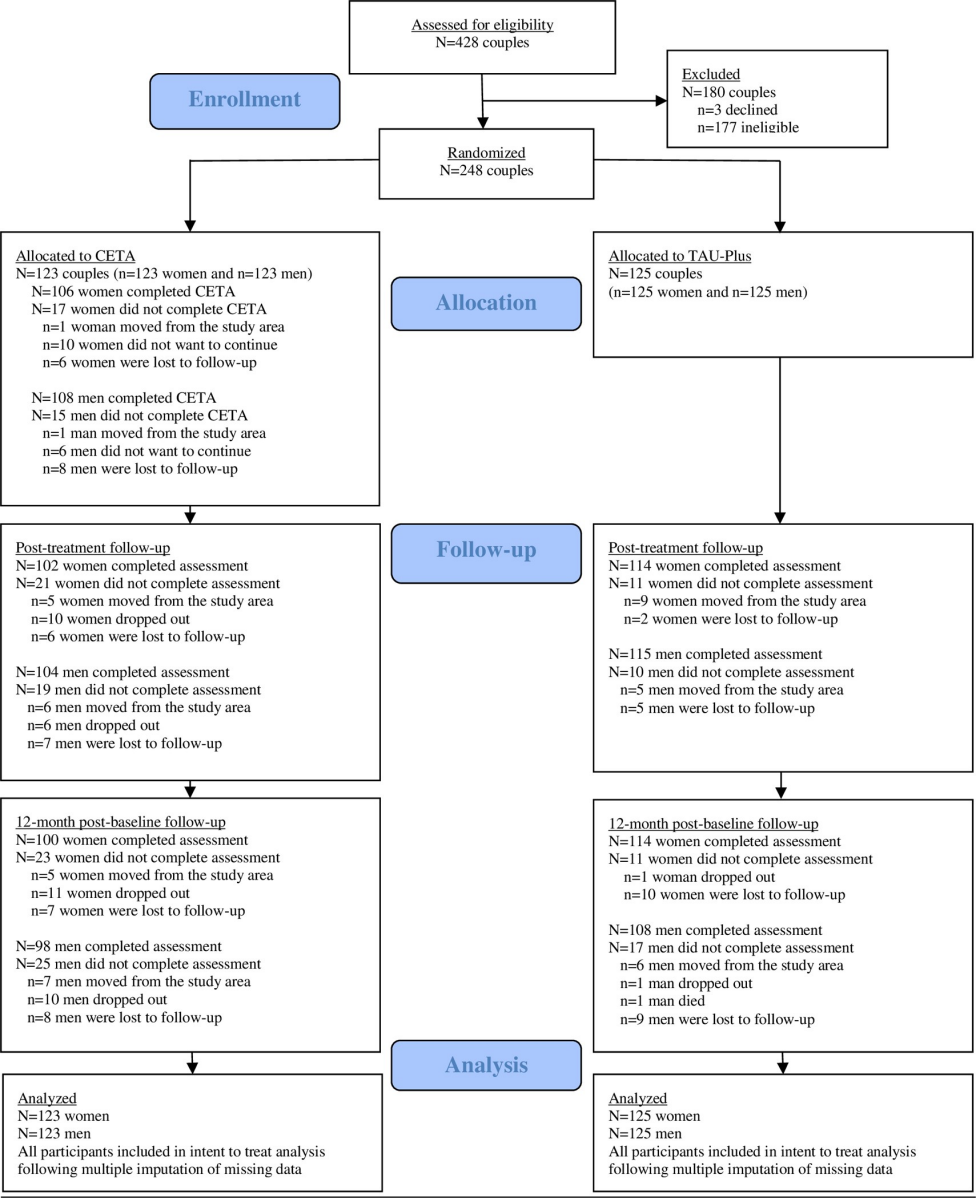
Trial flow diagram. CETA, Common Elements Treatment Approach; TAU-Plus, treatment as usual plus safety checks.

Enrollment of study participants was conducted between May 23, 2016, and December 17, 2016. By the end of enrollment, 123 couples were randomized to CETA and 125 to TAU-Plus. Eighty-six percent of women (*n* = 106) and 88% of men (*n =* 108) successfully completed CETA. Retention in the study was >80% for both women and men at the 12-month post-baseline visit. The CONSORT flow diagram is included as Fig 1.

Table 1 provides characteristics of the study sample at baseline. The sample skewed younger in age, with a majority of women (*n =* 164; 66%) and plurality of men (*n =* 121; 49%) 35 years of age or younger. Only 5% (*n =* 12) of women and 9% (*n =* 22) of men were formally employed. Both women and men reported high rates of experienced trauma, with a mean number of traumatic event types experienced in their lifetime greater than 5.

**Table 1:** Baseline characteristics of study sample.

Characteristic	CETA (*n =* 123)	TAU-Plus (*n =* 125)
**Age (years)**		
*Females*		
18–25	28 (23%)	37 (30%)
26–35	56 (46%)	43 (34%)
36–45	24 (19%)	25 (20%)
46–55	11 (9%)	14 (11%)
56–65	4 (3%)	3 (2%)
66+	0 (0%)	2 (2%)
Missing	0 (0%)	1 (1%)
*Males*		
18–25	18 (15%)	9 (7%)
26–35	41 (33%)	53 (43%)
36–45	39 (32%)	35 (28%)
46–55	18 (15%)	19 (15%)
56–65	5 (4%)	6 (5%)
66+	2 (1%)	3 (2%)
**Education**		
*Females*		
None	30 (24%)	28 (22%)
Some primary	53 (43%)	53 (42%)
Completed primary	21 (17%)	25 (20%)
Completed secondary	7 (6%)	12 (10%)
Completed higher than secondary	2 (2%)	2 (2%)
Other	10 (8%)	5 (4%)
*Males*		
None	19 (15%)	11 (9%)
Some primary	40 (33%)	51 (41%)
Completed primary	26 (21%)	24 (19%)
Completed secondary	28 (23%)	31 (25%)
Completed higher than secondary	4 (3%)	5 (4%)
Other	6 (5%)	3 (2%)
**Employment**		
*Females*		
Formally employed	9 (7%)	3 (2%)
Informally employed	23 (19%)	19 (15%)
Part-time employed	20 (16%)	28 (23%)
Unemployed and looking for work	59 (48%)	66 (53%)
Unemployed and not looking for work	12 (10%)	9 (7%)
*Males*		
Formally employed	14 (11%)	8 (7%)
Informally employed	36 (29%)	28 (22%)
Part-time employed	28 (23%)	23 (18%)
Unemployed and looking for work	42 (34%)	59 (47%)
Unemployed and not looking for work	3 (3%)	7 (6%)
**Relationship status**		
Spouse	47 (38%)	45 (36%)
Dating but not living together	9 (7%)	7 (6%)
Dating and living together	48 (39%)	61 (49%)
Other	19 (16%)	12 (9%)
**Physical disability**		
*Females*	83 (68%)	84 (67%)
*Males*	74 (61%)	66 (53%)
**Living with HIV**		
*Females*	55 (45%)	46 (37%)
*Males*	36 (30%)	30 (24%)
**Number of trauma types experienced**		
*Females*	7.7 (5.5)	6.7 (4.6)
*Males*	6.6 (5.3)	6.0 (5.0)
**Days between baseline and post-treatment assessment**		
*Females*	168.7 (75.8)	187.9 (44.3)
*Males*	168.4 (59.0)	192.8 (49.4)
**Days between baseline and 12-month post-baseline assessment**		
*Females*	389.9 (27.9)	383.9 (24.4)
*Males*	391.2 (32.3)	384.3 (30.3)

Data are *n* (%) or mean (SD).

CETA, Common Elements Treatment Approach; TAU-Plus, treatment as usual plus safety checks.

The DSMB interim analysis conducted at the 12-month post-baseline assessment with non-imputed data on the SVAWS physical/sexual violence subscale found a treatment effect expressed as a Cohen’s *d* effect size for CETA of 0.51 (difference in mean change = −8.5, 95% CI −13.8 to −3.3, *p* = 0.002). Based on this result, the DSMB made a recommendation to stop the trial early and offer CETA to control participants. Therefore, results presented below are for the post-treatment and 12-month post-baseline assessments only.

Table 2 presents the intervention effect for IPV outcomes following multiple imputation. For the primary outcome, SVAWS physical/sexual violence subscale, the treatment effect of CETA was 0.67 (difference in mean change = −11.3, 95% CI −16.7 to −5.8, *p* < 0.001) at the post-treatment assessment and 0.49 (difference in mean change = −8.2, 95% CI −14.9 to −1.5, *p* = 0.02) at the 12-month post-baseline assessment.

**Table 2:** Intervention effect of CETA on violence outcomes.

Outcome	CETA (*n* = 123)		TAU-Plus (*n* = 125)		Between-group treatment effect	
	Mean or *n* (%) (95% CI)	Mean change from baseline or relative risk (95% CI) *p*-value	Mean or *n* (%) (95% CI)	Mean change from baseline or relative risk (95% CI) *p*-value	Difference in mean change (95% CI) *p*-value	Cohen’s *d*
***Continuous variables***						
**SVAWS physical/sexual violence subscale (α = 0.92)**						
Baseline	65.2	—	61.8	—	—	—
	(62.0 to 68.3)		(60.4 to 63.2)			
End of treatment	38.6	−26.5	46.6	−15.2	−11.3	0.67
	(34.5 to 42.8)	(−30.5 to −22.5)	(42.7 to 50.5)	(−18.8 to −11.6)	(−16.7 to −5.8)	
		**<0.001**		**<0.001**	**<0.001**	
12-month	41.9	−23.2	46.8	−15.0	−8.2	0.49
	(37.6 to 46.2)	(−27.1 to −19.4)	(41.1 to 52.4)	(−20.6 to −9.5)	(−14.9 to −1.5)	
		**<0.001**		**<0.001**	**0.02**	
**SVAWS threatened violence subscale (α = 0.90)**						
Baseline	46.7	—	46.1	—	—	—
	(43.8 to 49.6)		(44.9 to 47.1)			
End of treatment	27.9	−18.8	33.5	−12.6	−6.2	0.49
	(24.6 to 31.2)	(−21.5 to −16.1)	(32.2 to 34.7)	(−13.6 to −11.5)	(−9.2 to −3.3)	
		**<0.001**		**<0.001**	**<0.001**	
12-month	29.7	−17.0	33.2	−12.9	−4.2	0.33
	(26.2 to 31.1)	(−20.1 to −14.0)	(30.9 to 35.4)	(−15.4 to −10.4)	(−8.0 to −0.3)	
		**<0.001**		**<0.001**	**0.04**	
***Binary variables***						
**Any physical violence experience—F**						
Baseline	98 (80%)	—	96 (77%)	—	—	
	(90 to 108)		(90 to 104)			
End of treatment	43 (35%)	0.43	63 (50%)	0.65	0.66	
	(33 to 55)	(0.34 to 0.56)	(56 to 69)	(0.61 to 0.70)	(0.51 to 0.87)	
		**<0.001**		**<0.001**	**0.003**	
12-month	47 (38%)	0.47	60 (48%)	0.63	0.75	
	(42 to 77)	(0.37 to 0.61)	(51 to 70)	(0.56 to 0.69)	(0.57 to 0.99)	
		**<0.001**		**<0.001**	**0.045**	
**Any physical violence perpetration—M**						
Baseline	98 (80%)	—	94 (75%)	—−	—	
	(93 to 105)		(86 to 103)			
End of treatment	31 (25%)	0.31	68 (54%)	0.73	0.43	
	(21 to 47)	(0.22 to 0.45)	(64 to 71)	(0.67 to 0.79)	(0.30 to 0.63)	
		**<0.001**		**<0.001**	**<0.001**	
12-month	47 (38%)	0.48	61 (49%)	0.66	0.73	
	(37 to 58)	(0.38 to 0.59)	(51 to 73)	(0.54 to 0.79)	(0.54 to 0.98)	
		**<0.001**		**<0.001**	**0.04**	
**Any sexual violence experience—F**						
Baseline	101 (82%)	—	88 (70%)	—	—	
	(89 to 113)		(80 to 95)			
End of treatment	43 (35%)	0.43	65 (52%)	0.74	0.58	
	(34 to 55)	(0.35 to 0.54)	(56 to 69)	(0.68 to 0.80)	(0.46 to 0.74)	
		**<0.001**		**<0.001**	**<0.001**	
12-month	44 (36%)	0.44	59 (47%)	0.68	0.65	
	(32 to 60)	(0.35 to 0.55)	(51 to 69)	(0.55 to 0.83)	(0.48 to 0.88)	
		**<0.001**		**<0.001**	**0.005**	
**Any sexual violence perpetration—M**						
Baseline	64 (52%)	—	54 (43%)	—	—	
	(55 to 75)		(48 to 60)			
End of treatment	27 (22%)	0.42	53 (42%)	0.99	0.42	
	(18 to 38)	(0.30 to 0.59)	(48 to 59)	(0.89 to 1.1)	(0.30 to 0.60)	
		**<0.001**		0.86	**<0.001**	
12-month	37 (30%)	0.58	45 (36%)	0.84	0.68	
	(27 to 52)	(0.40 to 0.82)	(36 to 56)	(0.70 to 0.99)	(0.46 to 1.0)	
		**0.002**		**0.04**	0.06	

α = Cronbach’s alpha for internal reliability. Estimates for mean, SD, mean change from baseline, difference in mean change, risk percent, RR, and ratio of relative risk are based on predicted values from mixed effects models. For binary outcomes, *N*s are calculated based on predicted percent. All participants were included in the analysis following multiple imputation of missing data. Cohen’s *d* effect size was calculated by dividing the predicted difference in mean change from the mixed effects model by the pooled baseline SD. Within-group RRs represent the change in risk from baseline to each post-baseline assessment. RRs < 1 indicate a reduction in risk. The ratio of RRs is the exponentiated group by time interaction term and represents the ratio of the CETA RR to the TAU-Plus RR. Between-group ratio of RRs < 1 indicates a greater reduction in risk from baseline to follow-up in the CETA group compared to the TAU-Plus group. All models included fixed effects of treatment arm, time, and the interaction term of treatment × time as well as random effects of participant ID and counselor ID. Additional fixed effect demographic variables were included as covariates if they differed meaningfully at baseline between the treatment groups or if the variable predicted change in the outcome over time. Specific variables included in each model are listed in S1 Table. Baseline and 12-month post-baseline assessments had an IPV reference period of the past 12 months; the post-treatment assessment had a reference period of the past 3 months. Bold indicates significant *p*-value.

CETA, Common Elements Treatment Approach; F, female report of experiencing recent violence; IPV, intimate partner violence; M, male report of perpetrating recent violence; RR, relative risk; SVAWS, Severity of Violence Against Women Scale; TAU-Plus, treatment as usual plus safety checks.

The result of the sensitivity sub-analysis for the primary SVAWS physical/sexual violence outcome—which compared CETA participants who received only individually delivered CETA (*n =* 83) (i.e., those who received any group therapy sessions were dropped from the analysis) to TAU-Plus participants (*n =* 125)—was similar to the intent-to-treat analysis at the post-treatment assessment (*d =* 0.67, difference in mean change = −11.0, 95% CI −17.5 to −4.6, *p* = 0.001) and the 12-month post-baseline assessment (*d* = 0.51, difference in mean change = −8.4, 95% CI −16.0 to −0.68, *p* = 0.03).

**Fig 2:**
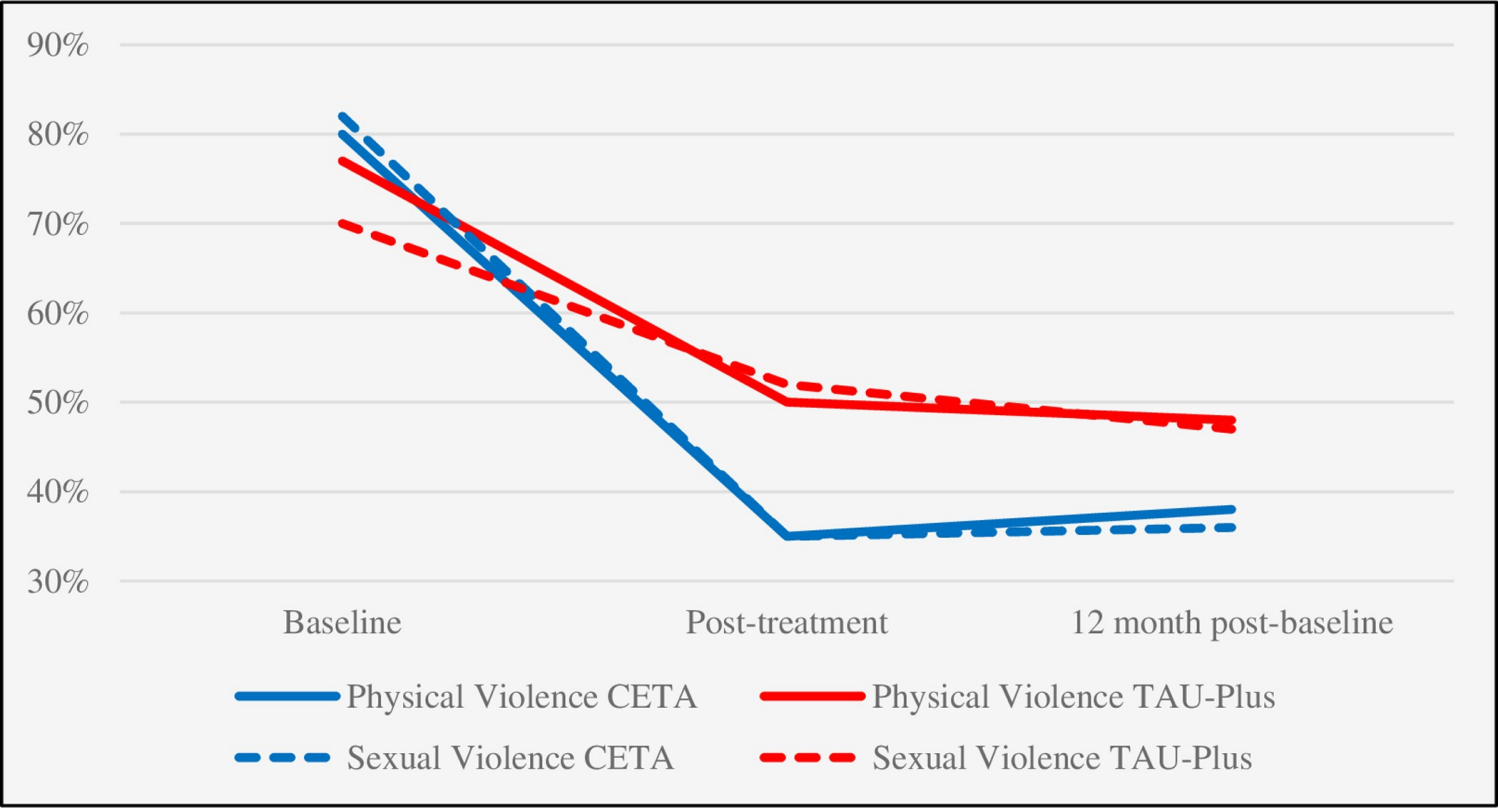
Change in female reporting of recent physical and sexual violence among those receiving CETA and TAU-Plus. The difference in change from baseline to post-treatment and from baseline to 12 months post-baseline between the groups was statistically significant for both physical and sexual violence (*p* < 0.05). Baseline and 12-month post-baseline assessments had an intimate partner violence reference period of the past 12 months; the post-treatment assessment had a reference period of the past 3 months. CETA, Common Elements Treatment Approach; TAU-Plus, treatment as usual plus safety checks.

Significant treatment effects for CETA were also observed for the SVAWS threatened violence subscale (*d* = 0.49, difference in mean change = −6.2, 95% CI −9.2 to −3.3, *p* < 0.001 post-treatment, and *d* = 0.33, difference in mean change = −4.2, 95% CI −8.0 to −0.3, *p* = 0.04, at 12-months post-baseline) and 3 out of the 4 binary violence outcomes (Fig 2). Female reporting of any past-year physical IPV reduced from 80% (*n =* 98) at baseline to 38% (*n =* 47) at 12 months post-baseline among women who were randomized to CETA, compared to a reduction from 77% (*n =* 96) to 48% (*n =* 60) among women randomized to TAU-Plus, a between-group RR of 0.75 (95% CI 0.57 to 0.99, *p* = 0.045). Significant between-group RRs at the 12-month assessment were also found for male report of physical violence perpetration (RR 0.73, 95% CI 0.54 to 0.98, *p* = 0.04) and female report of sexual violence experience (RR 0.65, 95% CI 0.48 to 0.74, *p* = 0.005). The effect for male report of sexual violence perpetration was large and reached borderline statistical significance (RR 0.68, 95% CI 0.46, 1.0, *p* = 0.06).

**Fig 3:**
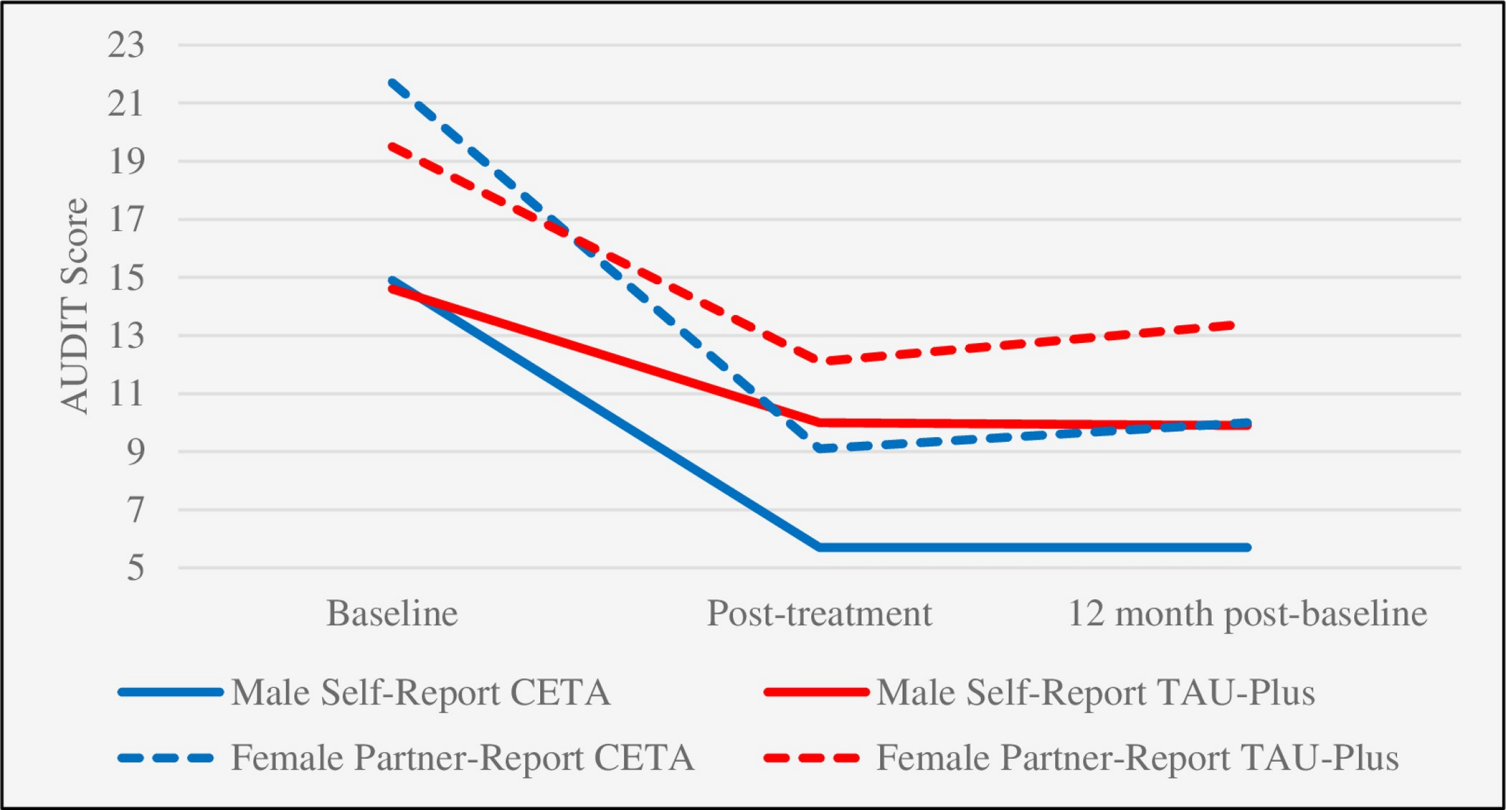
Change in male drinking among those receiving CETA and TAU-Plus as self-reported by the male and partner-reported by the female on AUDIT. The difference in change from baseline to post-treatment and from baseline to 12 months post-baseline between the groups was statistically significant for both male self-report and female partner-report (*p* < 0.05). AUDIT, Alcohol Use Disorders Identification Test; CETA, Common Elements Treatment Approach; TAU-Plus, treatment as usual plus safety checks.

CETA had significant treatment effects on male alcohol misuse (Table 3 and Fig 3). The effect size for male self-reported AUDIT score was 0.43 (difference in mean change = −4.5, 95% CI −6.2 to −2.2, *p* < 0.001) at the 12-month post-baseline assessment and was 0.59 (difference in mean change = −5.6, 95% CI −8.5 to −2.8, *p* < 0.001) for the female partner-reported AUDIT score. Treatment effects for female drinking were smaller: the effect size was 0.28 (difference in mean change = −3.1, 95% CI −5.3 to −0.99, *p* = 0.004) for female self-reported AUDIT score and 0.21 (difference in mean change = −1.9, 95% CI −3.9 to 0.1, *p* = 0.07) for male partner-reported AUDIT score.

**Table 3:** Intervention effect of CETA on alcohol outcomes.

AUDIT score	CETA (*n* = 123)		TAU-Plus (*n* = 125)		Between-group treatment effect	
	Mean (95% CI)	Mean change from baseline (95% CI) *p*-value	Mean (95% CI)	Mean change from baseline	Difference in mean change (95% CI) *p*-value	Cohen’s *d*
				(95% CI)		
				*p*-value		
**Male self-report (α = 0.85)**						
Baseline	14.9	—	14.6	—	—	—
	(13.3 to 16.4)		(13.8 to 15.4)			
End of treatment	5.7	−9.2	10.0	−4.5	−4.7	0.45
	(4.1 to 7.3)	(−11.7 to −6.6)	(8.8 to 11.3)	(−5.7 to −3.4)	(−7.5 to −1.8)	
		**<0.001**		**<0.001**	**<0.001**	
12-month	5.7	−9.2	9.9	−4.7	−4.5	0.43
	(3.7 to 7.7)	(−11.3 to −7.1)	(8.7 to 11.1)	(−6.9 to −2.2)	(−6.9 to −2.2)	
		**<0.001**		**<0.001**	**<0.001**	
**Female partner-report (α = 0.80)**						
Baseline	21.7	—	19.5	—	—	—
	(19.9 to 23.6)		(18.9 to 20.1)			
End of treatment	9.1	−12.7	12.1	−7.4	−5.3	0.56
	(6.9 to 11.2)	(−15.2 to −10.1)	(10.7 to 13.5)	(−8.5 to −6.3)	(−8.0 to −2.5)	
		**<0.001**		**<0.001**	**<0.001**	
12-month	10.0	−11.8	13.4	−6.1	−5.6	0.59
	(7.9 to 12.0)	(−14.3 to −9.3)	(11.7 to 15.1)	(−7.4 to −4.8)	(−8.5 to −2.8)	
		**<0.001**		**<0.001**	**<0.001**	
**Female self-report (α = 0.87)**						
Baseline	11.8	—	9.6	—	—	—
	(9.9 to 13.6)		(6.9 to 12.4)			
End of treatment	4.5	−7.2	5.3	−4.3	−2.9	0.26
	(2.6 to 6.4)	(−9.7 to −4.8)	(2.6 to 8.0)	(−5.4 to −3.2)	(−5.6 to −0.2)	
		**<0.001**		**<0.001**	**0.03**	
12-month	5.7	−6.0	6.7	−2.9	−3.1	0.28
	(3.7 to 7.8)	(−7.6 to −4.5)	(5.2 to 8.3)	(−4.4 to −1.4)	(−5.3 to −0.99)	
		**<0.001**		**<0.001**	**0.004**	
**Male partner-report (α = 0.78)**						
Baseline	9.9	—	9.0	—	—	—
	(8.2 to 11.6)		(7.4 to 10.6)			
End of treatment	5.7	−4.2	7.0	−2.0	−2.2	0.24
	(4.0 to 7.4)	(−6.5 to −1.9)	(5.7 to 8.4)	(−3.0 to −0.9)	(−4.7 to 0.3)	
		**<0.001**		**<0.001**	0.08	
12-month	6.2	−3.6	7.2	−1.8	−1.9	0.21
	(4.5 to 8.0)	(−5.1 to −2.2)	(5.9 to 8.5)	(−3.1 to −0.5)	(−3.9 to 0.1)	
		**<0.001**		**0.008**	0.07	

α = Cronbach’s alpha for internal reliability. Estimates for mean, SD, mean change from baseline, and difference in mean change are based on predicted values from mixed effects models. Cohen’s *d* effect size was calculated by dividing the predicted difference in mean change from the mixed effects model by the pooled baseline SD. All models included fixed effects of treatment arm, time, and the interaction term of treatment × time, as well as random effects of participant ID and counselor ID. Additional fixed effect demographic variables were included as covariates if they differed meaningfully at baseline between the treatment groups or if the variable predicted change in the outcome over time. Specific variables included in each model are listed in S1 Table. Bold indicates significant *p*-value.

AUDIT, Alcohol Use Disorders Identification Test; CETA, Common Elements Treatment Approach; TAU-Plus, treatment as usual plus safety checks.

The results of the sensitivity analysis in which the baseline outcome values were included as fixed effects in the models are displayed in S2 Table. There were no significant differences between the results of the primary models and the those of the sensitivity analysis.

No adverse events related to the intervention were reported to the study team. A total of 279 high-risk events (suicidal ideation, homicidal ideation, current IPV, and/or child abuse) were reported during baseline assessment, treatment, and/or monitoring sessions: 158 clients reported suicidal ideation (males, *n =* 79; females, *n =* 63), 112 clients reported active IPV (males, *n =* 1; females, *n =* 84), and 7 clients reported active homicidal ideation (males, *n =* 4; females, *n =* 3). All cases were handled using the approved safety protocol.

## Discussion

Although there are multiple studies on primary prevention and economic approaches for IPV in LMICs [50,51], this study is one of the first to test an evidence-based mental/behavioral health secondary prevention intervention. This RCT showed that CETA was more effective than TAU-Plus in reducing IPV and hazardous alcohol use among high-risk couples in Zambia. Our findings on IPV are strengthened by the general consistency of the statistical significance of reductions observed in multiple forms of violence (physical and sexual), as reported by both men (perpetrating) and women (experiencing), and as measured by 2 different instruments. In addition, the results suggest that the effects of CETA on alcohol misuse and IPV were sustained for at least 1 year following treatment commencement.

The design of this trial purposefully utilized a multifaceted approach to address violence and related psychosocial problems (e.g., relationship skills) directly by delivering CBT-based skills including safety planning, and also indirectly by treating alcohol misuse, one of the strongest IPV risk factors. The clinically significant effects in the study are likely related to the multifaceted approach of CETA. A WHO global status report on violence discusses 7 “best buy” issues to address for the prevention of (6 strategies) and response to (1 strategy) violence in LMICs based on the assessment that each could impact a range of violence types [52]. CETA (as a single program) is equipped to address 4 of these issues: family relationships, life skills, alcohol misuse, and treatment for survivors. CETA elements are CBT-based and are therefore designed to be “life skills” with the goal of changing behaviors and thoughts, and helping individuals feel better not just in the short term, but over time. This skill-based approach is likely linked to the sustainment of treatment effects 1 year after baseline.

In addition to the encouraging findings on IPV, the study is one of the first in LMICs to demonstrate that a lay counselor–delivered, evidence-based substance use element was effective in reducing hazardous alcohol use. Our findings are in line with those of Papas and colleagues, who found short-term significant CBT treatment effects for alcohol misuse among adults with HIV in Kenya [53]. Our results extend those to show that treatment effects were sustained for at least 1 year following the beginning of CETA treatment. This is significant as rates of relapse are typically high among substance use disorders [54]. Notably, for male alcohol use, men underreported their own consumption relative to the women’s report of men’s drinking, but significant CETA effects were observed regardless of reporter.

Although CETA treatment demonstrated strong effectiveness in reducing IPV, there was also a significant reduction of violence in the control group. This highlights the potential efficacy for some couples of a safety check-in approach, which could require less time and resources than a more comprehensive multi-problem approach like CETA. This study was not designed to evaluate the effectiveness of the safety check-in approach, and important questions remain as to which populations would benefit from safety checks alone, whether the checks alone would have lasting effects given that they do not provide clients with skills, and whether the checks would need to continue indefinitely, which would require significant resources. Future studies are needed to evaluate the cost-effectiveness differentials of a safety-alone approach to IPV. At minimum, we recommend that safety check-ins for suicide should be included in all programing and studies on IPV prevention and treatment [37].

This study had limitations. First, given the high-risk nature of the study sample, we were not able to design a study with a true control condition. Ethically, our control condition had to be augmented with weekly safety checks with the family for high-risk situations including suicide, homicide, and/or abuse to the point of physical danger. Additionally, we acknowledge the possibility of contamination in that it is possible couples in the study communities who were in different arms of the trial spoke to each other about the content of the intervention. If the safety checks and/or possible contamination led to reductions in IPV or alcohol misuse among control participants, this would make our CETA treatment effect findings conservative. Second, the study was possibly underpowered for analyses of the secondary outcomes, particularly the binary IPV variables. Third, like most studies on IPV and alcohol misuse, this study relied on self-report assessment of outcomes, which is subject to social desirability and recall biases [55]. Relatedly, the WHO-derived IPV measure has not previously been validated in Zambia to our knowledge. However, the similarity in findings across the IPV measures (SVAWS and female- and male-reported WHO-derived IPV items) collectively support the finding of CETA effectiveness. Future investigations would benefit from the use of biomarkers when feasible [56]. Fourth, although retention in CETA was high overall, there was a greater number of participants who were categorized as “drop-outs” (not including those who were lost to follow-up or moved from the study area) at the 12-month assessment among those randomized to CETA (*n =* 21) than among those randomized to TAU-Plus (*n =* 2). We believe the difference is likely attributable to the additional study burden (i.e., time, travel) for participants in the CETA arm versus the control arm. All participants were included in the intent-to-treat analysis, and we believe that the use of multiple imputation procedures was appropriate for handling the missing data.

Currently, CETA is the only modular, flexible, multi-problem, transdiagnostic approach delivered by lay providers that has multiple RCT evaluations in LMICs. Adding to the existing evidence showing CETA’s effectiveness on a range of mental and behavioral health issues [30–32], this trial provides evidence of the broader effectiveness of CETA in addressing social issues of IPV and alcohol misuse. Collectively, the evidence suggests that this transdiagnostic model can more effectively, efficiently, and economically address naturally occurring comorbidity in the human population to reduce the mental and behavioral health treatment gap in LMICs than interventions that focus on a single problem. CETA’s application to reduce IPV as a secondary prevention approach represents a novel contribution to the violence reduction field, which has primarily focused on community mobilization and primary prevention methods [50]. Given that IPV is such a complex social problem, future evaluations would benefit from combining primary, secondary, and tertiary prevention approaches to enhance effectiveness, such as combining CETA with effective economic interventions [51] and potentially higher level psychological and/or psychiatric care when available and indicated (e.g., pharmacological approaches in cases of alcohol dependence).

## Supporting information

S1 Appendix Study questionnaire. (DOCX)

S2 Appendix CONSORT checklist. (DOC)

S1 Table Covariates included in models. (DOCX)

S2 Table Results of sensitivity analysis. (DOCX)

## Abbreviations

ACASI: audio computer-assisted self-interviewing
AUDIT: Alcohol Use Disorders Identification Test
CBT: cognitive behavioral therapy
CETA: Common Elements Treatment Approach
DSMB: Data and Safety Monitoring Board
IPV: intimate partner violence
LMICs: low- and middle-income countries
RCT: randomized controlled trial
RR: relative risk
SVAWS: Severity of Violence Against Women Scale
TAU-Plus: treatment as usual plus safety checks
