# Supplementary material for: Effectiveness of the Common Elements Treatment Approach (CETA) in reducing intimate partner violence and hazardous alcohol use in Zambia (VATU): A randomized controlled trial
Source: PLoS Med. 2020 Apr 17;17(4):e1003056. doi: 10.1371/journal.pmed.1003056 (PMC7164585; doi:10.1371/journal.pmed.1003056)
Supplement: S1 Appendix — (DOCX) [file pmed.1003056.s001.docx]

**S1. Appendix:** Study Questionnaire

**Severity of Violence Against Women Scale (SVAWS)**

| How often has your partner: | Never | Once | A Few Times | Many Times | Don’t Know | Refused |
| --- | --- | --- | --- | --- | --- | --- |
| 2.1. Hit or kicked a wall, door, or furniture | 1 | 2 | 3 | 4 | -8 | -9 |
| 2.2. Threw, smashed, or broke an object | 1 | 2 | 3 | 4 | -8 | -9 |
| 2.3. Drove dangerously with you in the car | 1 | 2 | 3 | 4 | -8 | -9 |
| 2.4. Threw an object at you | 1 | 2 | 3 | 4 | -8 | -9 |
| 2.5. Shook or pointed a finger at you | 1 | 2 | 3 | 4 | -8 | -9 |
| 2.6. Made threatening gestures or faces at you | 1 | 2 | 3 | 4 | -8 | -9 |
| 2.7. Shook a fist at you | 1 | 2 | 3 | 4 | -8 | -9 |
| 2.8. Acted like a bully toward you | 1 | 2 | 3 | 4 | -8 | -9 |
| 2.9. Destroyed something belonging to you | 1 | 2 | 3 | 4 | -8 | -9 |
| 2.10. Threatened to harm or damage things you care about | 1 | 2 | 3 | 4 | -8 | -9 |
| 2.11. Threatened to destroy property | 1 | 2 | 3 | 4 | -8 | -9 |
| 2.12. Threatened someone you care about | 1 | 2 | 3 | 4 | -8 | -9 |
| 2.13. Threatened to hurt you | 1 | 2 | 3 | 4 | -8 | -9 |
| 2.14. Threatened to kill himself | 1 | 2 | 3 | 4 | -8 | -9 |
| 2.15. Threatened to kill you | 1 | 2 | 3 | 4 | -8 | -9 |
| 2.16. Threatened you with a weapon | 1 | 2 | 3 | 4 | -8 | -9 |
| 2.17. Threatened you with a heavy stick or object | 1 | 2 | 3 | 4 | -8 | -9 |
| 2.18. Acted like he wanted to kill you | 1 | 2 | 3 | 4 | -8 | -9 |
| 2.19. Threatened you with a knife or gun | 1 | 2 | 3 | 4 | -8 | -9 |
| 2.20. Held you down, pinning you in place | 1 | 2 | 3 | 4 | -8 | -9 |

| How often has your partner: | Never | Once | A Few Times | Many Times | Don’t Know | Refused |
| --- | --- | --- | --- | --- | --- | --- |
| 2.21. Pushed or shoved you | 1 | 2 | 3 | 4 | -8 | -9 |
| 2.22. Grabbed you suddenly or forcefully | 1 | 2 | 3 | 4 | -8 | -9 |
| 2.23. Shook or roughly handled you | 1 | 2 | 3 | 4 | -8 | -9 |
| 2.24. Scratched you | 1 | 2 | 3 | 4 | -8 | -9 |
| 2.25. Pulled your hair | 1 | 2 | 3 | 4 | -8 | -9 |
| 2.26. Twisted your arm | 1 | 2 | 3 | 4 | -8 | -9 |
| 2.27. Spanked you | 1 | 2 | 3 | 4 | -8 | -9 |
| 2.28. Bit you | 1 | 2 | 3 | 4 | -8 | -9 |
| 2.29. Slapped you with the palm of his hand | 1 | 2 | 3 | 4 | -8 | -9 |
| 2.30. Slapped you with the back of his hand | 1 | 2 | 3 | 4 | -8 | -9 |
| 2.31. Slapped you around your face and head | 1 | 2 | 3 | 4 | -8 | -9 |
| 2.32. Hit you with an object | 1 | 2 | 3 | 4 | -8 | -9 |
| 2.33. Punched you | 1 | 2 | 3 | 4 | -8 | -9 |
| 2.34. Kicked you | 1 | 2 | 3 | 4 | -8 | -9 |
| 2.35. Stomped on you | 1 | 2 | 3 | 4 | -8 | -9 |
| 2.36. Suffocated/strangled you | 1 | 2 | 3 | 4 | -8 | -9 |
| 2.37. Burned you with something | 1 | 2 | 3 | 4 | -8 | -9 |
| 2.38. Used a club-like object on you | 1 | 2 | 3 | 4 | -8 | -9 |
| 2.39. Beat you up | 1 | 2 | 3 | 4 | -8 | -9 |
| 2.40. Used a knife or gun on you | 1 | 2 | 3 | 4 | -8 | -9 |
| 2.41. Demanded sex whether you wanted it or not | 1 | 2 | 3 | 4 | -8 | -9 |
| 2.42. Made you have oral sex against your will | 1 | 2 | 3 | 4 | -8 | -9 |
| 2.43. Made you have sexual intercourse against your will | 1 | 2 | 3 | 4 | -8 | -9 |
| 2.44. Physically forced you to have sex | 1 | 2 | 3 | 4 | -8 | -9 |
| 2.45. Made you have anal sex against your will | 1 | 2 | 3 | 4 | -8 | -9 |
| 2.46. Used an object on you in a sexual way | 1 | 2 | 3 | 4 | -8 | -9 |

**Alcohol Use Disorders Identification Test (AUDIT): Partner-Report**

Please think about the alcohol use of your *partner* in the following questions.

| 3.1 How often did your partner have a drink containing alcohol? | Never | 0 |
| --- | --- | --- |
|  | Once a month | 1 |
|  | 2 to 4 times per month | 2 |
|  | 2 to 3 times per week | 3 |
|  | 4 or more times per week | 4 |
|  | Don’t know | -8 |
|  | Refused | -9 |
| 3.2 How many drinks containing alcohol did your partner have on a typical day when your partner was drinking? | 1 or 2 | 0 |
|  | 3 or 4 | 1 |
|  | 5 or 6 | 2 |
|  | 7, 8, or 9 | 3 |
|  | 10 or more | 4 |
|  | Don’t know | -8 |
|  | Refused | -9 |
| 3.3 How often did your partner have six or more drinks on one occasion? | Never | 0 |
|  | Less than monthly | 1 |
|  | Monthly | 2 |
|  | Weekly | 3 |
|  | Daily or almost daily | 4 |
|  | Refused | -9 |
|  | Don’t know | -8 |

| 3.4 How often during the last year has your partner  not been able to stop drinking once your partner  had started? | Never | 0 |
| --- | --- | --- |
|  | Less than monthly | 1 |
|  | Monthly | 2 |
|  | Weekly | 3 |
|  | Daily or almost daily | 4 |
|  | Refused | -9 |
|  | Don’t know | -8 |
| 3.5 How often during the last year has your partner failed to do what was normally expected from them because of their drinking? | Never | 0 |
|  | Less than monthly | 1 |
|  | Monthly | 2 |
|  | Weekly | 3 |
|  | Daily or almost daily | 4 |
|  | Refused | -9 |
|  | Don’t know | -8 |
| 3.6 How often during the last year did your partner need a first drink in the morning to get going after a heavy drinking session? | Never | 0 |
|  | Less than monthly | 1 |
|  | Monthly | 2 |
|  | Weekly | 3 |
|  | Daily or almost daily | 4 |
|  | Refused | -9 |
|  | Don’t know | -8 |

| 3.7 How often during the last year has your partner had a feeling of guilt or regret after drinking? | Never | 0 |
| --- | --- | --- |
|  | Less than monthly | 1 |
|  | Monthly | 2 |
|  | Weekly | 3 |
|  | Daily or almost daily | 4 |
|  | Refused | -9 |
|  | Don’t know | -8 |
| 3.8 How often during the last year has your partner been unable to remember what happened the night before because they had been drinking? | Never | 0 |
|  | Less than monthly | 1 |
|  | Monthly | 2 |
|  | Weekly | 3 |
|  | Daily or almost daily | 4 |
|  | Refused | -9 |
|  | Don’t know | -8 |
| 3.9 Have you or someone else been injured as a  result of your partner’s drinking? | No | 0 |
|  | Yes, but not in the last year | 2 |
|  | Yes, during the last year | 4 |
|  | Refused | -9 |
|  | Don’t know | -8 |

| 3.10 Has a relative or friend or a doctor or another health worker been concerned about your partner’s drinking or suggested your partner cut down? | No | 0 |
| --- | --- | --- |
|  | Yes, but not in the last year | 2 |
|  | Yes, during the last year | 4 |
|  | Refused | -9 |
|  | Don’t know | -8 |

**Alcohol Use Disorders Identification Test (AUDIT): Self-Report**

Please think about your *own* alcohol use in the following questions.

| 4.1 How often did you have a drink containing alcohol? | Never | 0 |
| --- | --- | --- |
|  | Once a month | 1 |
|  | 2 to 4 times per month | 2 |
|  | 2 to 3 times per week | 3 |
|  | 4 or more times per week | 4 |
|  | Don’t know | -8 |
|  | Refused | -9 |
| 4.2 How many drinks containing alcohol did you have on a typical day when you were drinking? | 1 or 2 | 0 |
|  | 3 or 4 | 1 |
|  | 5 or 6 | 2 |
|  | 7, 8, or 9 | 3 |
|  | 10 or more | 4 |
|  | Don’t know | -8 |
|  | Refused | -9 |
| 4.3 How often did you have six or more drinks on one occasion? | Never | 0 |
|  | Less than monthly | 1 |
|  | Monthly | 2 |
|  | Weekly | 3 |
|  | Daily or almost daily | 4 |
|  | Refused | -9 |
|  | Don’t know | -8 |

| 4.4 How often during the last year have you found  that you were not able to stop drinking once you  had started? | Never | 0 |
| --- | --- | --- |
|  | Less than monthly | 1 |
|  | Monthly | 2 |
|  | Weekly | 3 |
|  | Daily or almost daily | 4 |
|  | Refused | -9 |
|  | Don’t know | -8 |
| 4.5 How often during the last year have you failed to do what was normally expected from you  because of drinking? | Never | 0 |
|  | Less than monthly | 1 |
|  | Monthly | 2 |
|  | Weekly | 3 |
|  | Daily or almost daily | 4 |
|  | Refused | -9 |
|  | Don’t know | -8 |
| 4.6 How often during the last year have you needed a first drink in the morning to get yourself going after a heavy drinking session? | Never | 0 |
|  | Less than monthly | 1 |
|  | Monthly | 2 |
|  | Weekly | 3 |
|  | Daily or almost daily | 4 |
|  | Refused | -9 |
|  | Don’t know | -8 |

| 4.7 How often during the last year have you had a  feeling of guilt or regret after drinking? | Never | 0 |
| --- | --- | --- |
|  | Less than monthly | 1 |
|  | Monthly | 2 |
|  | Weekly | 3 |
|  | Daily or almost daily | 4 |
|  | Refused | -9 |
|  | Don’t know | -8 |
| 4.8 How often during the last year have you been  unable to remember what happened the night  before because you had been drinking? | Never | 0 |
|  | Less than monthly | 1 |
|  | Monthly | 2 |
|  | Weekly | 3 |
|  | Daily or almost daily | 4 |
|  | Refused | -9 |
|  | Don’t know | -8 |
| 4.9 Have you or someone else been injured as a  result of your drinking? | No | 0 |
|  | Yes, but not in the last year | 2 |
|  | Yes, during the last year | 4 |
|  | Refused | -9 |
|  | Don’t know | -8 |

| 4.10 Has a relative or friend or a doctor or another health worker been concerned about your drinking or suggested you cut down? | No | 0 |
| --- | --- | --- |
|  | Yes, but not in the last year | 2 |
|  | Yes, during the last year | 4 |
|  | Refused | -9 |
|  | Don’t know | -8 |

**WHO/What Works Violence Scale**

**Note:** for male participants, questions were modified to refer to IPV perpetration

| In the past 12 months… | Never | Once | Few | Many | Don’t Know | Refused |
| --- | --- | --- | --- | --- | --- | --- |
| 9.1. How many times has a current or previous husband or boyfriend ever slapped you or thrown something at you which could hurt you? | 0 | 1 | 2 | 3 | -8 | -9 |
| 9.2. How many times has a current or previous husband or boyfriend ever pushed or shoved you? | 0 | 1 | 2 | 3 | -8 | -9 |
| 9.3. How many times has a current or previous husband or boyfriend ever hit you with a fist or with something else which could hurt you? | 0 | 1 | 2 | 3 | -8 | -9 |
| 9.4. How many times has a current or previous husband or boyfriend ever kicked, dragged, beaten, choked or burnt you? | 0 | 1 | 2 | 3 | -8 | -9 |
| 9.5. How many times has a current or previous husband or boyfriend ever threatened to use or actually used a gun, knife or other weapon against you? | 0 | 1 | 2 | 3 | -8 | -9 |
| 9.6. How many times has a current or previous husband or boyfriend physically forced you to have sex when you did not want to? | 0 | 1 | 2 | 3 | -8 | -9 |
| 9.7. How many times have you had sex with a current or previous husband or boyfriend when you did not want to because you were afraid that he might become violent? | 0 | 1 | 2 | 3 | -8 | -9 |
| 9. How many times has a current or previous husband or boyfriend ever forced you to do something else sexual that you did not want to do? | 0 | 1 | 2 | 3 | -8 | -9 |
