# Supplementary material for: Effectiveness of the Common Elements Treatment Approach (CETA) in reducing intimate partner violence and hazardous alcohol use in Zambia (VATU): A randomized controlled trial
Source: PLoS Med. 2020 Apr 17;17(4):e1003056. doi: 10.1371/journal.pmed.1003056 (PMC7164585; doi:10.1371/journal.pmed.1003056)
Supplement: S1 Table — (DOCX) [file pmed.1003056.s003.docx]

All models included fixed effects of treatment arm, time, and interaction terms of treatment X time as well as random effects of participant ID and counselor ID. Additional fixed effect demographic variables were included as covariates if they differed meaningfully at baseline between the treatment groups or if the variable predicted change in the outcome over time. Specific variables included in each model are listed below:

| **S1 Table.** Covariates included in models | | |
| --- | --- | --- |
| **Model number** | **Outcome measure** | **Covariates included as fixed effects** |
| Model 1 | SVAWS physical/sexual violence subscale | Number of days between baseline and post-treatment assessment; age; number of trauma types experienced |
| Model 2 | SVAWS threatened violence subscale | Number of days between baseline and post-treatment assessment; age; number of trauma types experienced |
| Model 3 | WHO IPV: Any physical violence experienced (female report) | Number of days between baseline and post-treatment assessment |
| Model 4 | WHO IPV: Any physical violence perpetrated (male report) | Number of days between baseline and post-treatment assessment |
| Model 5 | WHO IPV: Any sexual violence experienced (female report) | Number of days between baseline and post-treatment assessment |
| Model 6 | WHO IPV: Any sexual violence perpetrated (male report) | Number of days between baseline and post-treatment assessment; number of trauma types experienced |
| Model 7 | AUDIT: male self-report | Number of days between baseline and post-treatment assessment; number of trauma types experienced; disability |
| Model 8 | AUDIT: female partner-report | Number of days between baseline and post-treatment assessment |
| Model 9 | AUDIT: female self-report | Number of days between baseline and post-treatment assessment |
| Model 10 | AUDIT: male partner-report | Number of days between baseline and post-treatment assessment; income; disability |
