# Supplementary material for: Effectiveness of the Common Elements Treatment Approach (CETA) in reducing intimate partner violence and hazardous alcohol use in Zambia (VATU): A randomized controlled trial
Source: PLoS Med. 2020 Apr 17;17(4):e1003056. doi: 10.1371/journal.pmed.1003056 (PMC7164585; doi:10.1371/journal.pmed.1003056)
Supplement: S2 Table — (DOCX) [file pmed.1003056.s004.docx]

| **S2 Table**. Results of sensitivity analysis:  Intervention effects of CETA on violence and alcohol outcomes at 12-month post-baseline after adjusting for baseline outcome values | | |
| --- | --- | --- |
|  | Between-group treatment effect | |
| ***Continuous outcomes*** | ***Difference in mean change***  ***(95% CI)***  ***p*** | **Cohen’s d** |
| SVAWS physical/sexual violence subscale | -8.2  (-14.9 to -1.5)  **.02** | 0.49 |
| SVAWS threatened violence subscale | -4.2  (-8.0 to -0.3)  **.04** | 0.33 |
| Male self-report AUDIT | -4.5  (-6.9 to -2.2)  **<.001** | 0.43 |
| Female partner-report AUDIT | -5.7  (-8.5 to -2.8)  **<.001** | 0.60 |
| Female self-report AUDIT | -3.1  (-5.2 to -0.9)  **<.01** | 0.28 |
| Male partner-report AUDIT | -1.9  (-3.9 to 0.12)  .07 | 0.21 |
| ***Binary outcomes*** | ***Ratio of relative risks***  ***95% CI***  ***p*** | |
| Any physical violence experience (female) | 0.75  (0.57 to 0.99)  **.045** | |
| Any physical violence perpetration (male) | 0.73  (0.54 to 0.98)  **.04** | |
| Any sexual violence experience (female) | 0.65  (0.48 to 0.88)  **<.01** | |
| Any sexual violence perpetration (male) | 0.68  (0.46 to 1.02)  .06 | |
| SVAWS=Severity of Violence Against Women Scale  Difference in mean change and ratio of relative risk are based on predicted values from mixed effects models. All participants were included in the analysis following multiple imputation of missing data.  Cohen’s *d* effect size is calculated by dividing the predicted difference in mean change from the mixed effects model by the pooled baseline SD.  The ratio of relative risks is the exponentiated group by time interaction term and represents the ratio of the CETA relative risk to the TAU-Plus relative risk. Between group ratio of relative risk <1 indicates a greater reduction in risk from baseline to follow-up in the CETA group compared to TAU-Plus.  All models included fixed effects of baseline outcome value, treatment arm, time, and interaction terms of treatment X time as well as random effects of participant ID and counselor ID. Additional fixed effect demographic variables were included as covariates if they differed meaningfully at baseline between the treatment groups or if the variable predicted change in the outcome over time. Specific variables included in each model are listed in S1 Table. | | |
